# Aggregation/dispersion transitions of T4 phage triggered by environmental ion availability

**DOI:** 10.1186/s12951-017-0266-5

**Published:** 2017-04-24

**Authors:** Bożena Szermer-Olearnik, Marek Drab, Mateusz Mąkosa, Maria Zembala, Jakub Barbasz, Krystyna Dąbrowska, Janusz Boratyński

**Affiliations:** 1 Laboratory of Biomedical Chemistry-“Neolek”. Hirszfeld Institute of Immunology and Experimental Therapy, Polish Academy of Sciences, R. Weigla 12, 53-114 Wroclaw, Poland; 2 USI, Unit of Nanostructural Bio-Interactions, Hirszfeld Institute of Immunology and Experimental Therapy, Polish Academy of Sciences, R. Weigla 12, 53-114 Wrocław, Poland; 3 Institute of Biology, Pedagogical University of Cracow, Podchorążych 2, 30-084 Cracow, Poland; 4 Institute of Catalysis and Surface Chemistry, Polish Academy of Sciences, Niezapominajek 8, 30-239 Cracow, Poland; 5 Bacteriophage Laboratory, Hirszfeld Institute of Immunology and Experimental Therapy, Polish Academy of Sciences, R. Weigla 12, 53-114 Wrocław, Poland; 6 Institute of Chemistry, Environmental Protection and Biotechnology, Jan Długosz University, 42-200 Częstochowa, Poland

## Abstract

**Background:**

Bacteriophage survives in at least two extremes of ionic environments: bacterial host *(high ionic*-*cytosol*) and that of soil (*low ionic*-*environmental water*). The impact of ionic composition in the micro- and macro-environments has not so far been addressed in phage biology.

**Results:**

Here, we discovered a novel mechanism of aggregation/disaggregation transitions by phage virions. When normal sodium levels in phage media (150 mM) were lowered to 10 mM, advanced imaging by scanning electron microscopy, atomic force microscopy and dynamic light scattering all revealed formation of viral packages, each containing 20–100 virions. When ionic strength was returned from low to high, the aggregated state of phage reversed to a dispersed state, and the change in ionic strength did not substantially affect infectivity of the phage. By providing the direct evidence, that lowering of the sodium ion below the threshold of 20 mM causes rapid aggregation of phage while returning Na^+^ concentration to the values above this threshold causes dispersion of phage, we identified a biophysical mechanism of phage aggregation.

**Conclusions:**

Our results implicate operation of group behavior in phage and suggest a new kind of quorum sensing among its virions that is mediated by ions. Loss of ionic strength may act as a trigger in an evolutionary mechanism to improve the survival of bacteriophage by stimulating aggregation of phage when outside a bacterial host. Reversal of phage aggregation is also a promising breakthrough in biotechnological applications, since we demonstrated here the ability to retain viable virion aggregates on standard micro-filters.

## Background

Bacteriophage virions belong to the environmental microbiome. They influence the bacterial equilibrium in terrestrial and aquatic environments [1–3]. They also belong to the microbiome of the human and animal intestine [4, 5]. T4 phage was originally derived from *Escherichia coli*, the leading marker of human contamination of water worldwide. Thus, *E. coli* is one of the most significant microbes of the human body and together with its phage, T4 has a vast effect on the human habitat.

In addition to their roles in modulating the micro-biosphere, bacteriophages have become powerful tools in research and development, particularly in biotechnology and medicine, where they hold promise for medical treatment of infections and cancer [6–8]. As with most of other nanoparticles, bacteriophages are also subject to small-size related problems in purification and scaling-up, both of which are critical for applications involving phage, where preserving delicate structure and protecting its biological activity is paramount [9].

Bacteriophages are part of a complex biological system of inter-order (virus/bacteria) relations, i.e. an interactome with associations operating across several scales of strength from weak to strong bonding, driven by predominantly non-linear ligand-receptor type of interactions [9, 10].

Effects of mono- and divalent alkali cations on the T2, T4 and T5 phage were tested as early as 1953, in elegant work by Lark and Adams [11]. The authors studied phage-killing effects of heat-inactivation at 50–70 °C and found that under such harsh conditions phage inactivation was prevented by alkali cations. Lithium, sodium, potassium and magnesium all had similar stabilizing effects on phage. Lowering cation concentration below 100 mM rendered phage sensitive to 50 °C-heat inactivation. Based on physicochemical kinetics and electron microscopy, the conclusion was drawn that ionic environment might affect the substructure of phage virion and that extreme salt concentrations (e.g. 4 M NaCl) could facilitate phage killing when followed by rapid dilution to induce osmotic shock. DNA loss from capsids by heat exposure was proposed as a mechanism of inactivation, along with protein denaturation, which would not be surprising at temperatures used for heat inactivation, 50–70 °C. Of the divalent cations studied, Mg and Ca, only magnesium exerted heat-stabilizing effects on phage. The impact of ionic strength has been less clear for divalent cations since their salts affect enzymatic activity of proteins. Thus, divalent alkali ions may be too complex for practical use. Therefore, investigators focused on monovalent alkali cations for future studies as more interesting subjects [11]. The work of Lark and Adams prompted us to investigate effects of alkali monovalent cations on phage. Since all monovalent alkali cations studied exerted similar effects in the studies of Lark and Adams, we chose to focus on sodium for our work.

Leibo and Mazur proposed utilizing the aggregation of phage on standard microfilters as far back as 1966 [12]. Aggregation of phage by salts with subsequent elimination by filtration has been proposed for virion elimination from water. To achieve this goal, Leibo and Mazur used harsh osmotic shock with 3 M NaCl in order to trigger DNA release and removal of virions from liquid media [12]. Phage filtration through induction of virion aggregation with salt was also addressed in the work of Furiga et al. in 2011 [13]. In their study, aggregation of phage (RNA phage M2) was recognized as a strong inactivating factor, which dramatically reduced infectivity of phage.

In contrast to studies outlined above, the phenomenon of phage aggregation has sporadically been reported to be associated with phage survival [10, 14–18]. For instance, MS2 bacteriophage aggregates were found to be more resistant to microenvironmental changes than their dispersed forms [19]. However, the interpretation of these results are complicated by lack of information on culture media. Colloidal, artificially-manipulated environments were generated in situ in bacterial plaques to limit thermal motion and preserve naturally occurring phage aggregation during life cycle in hosts; the used gel-like media could have had an impact on phage aggregation irrespective of ionic strength.

The importance of aggregation as an evolutionary mechanism designed to limit the effects of harsh environmental conditions on virions has been proposed previously [10]. However, ionic strength as regulator of aggregation/dispersion has not so far been fully addressed.

Evidence that phage is capable of sensing the ionic milieu was first provided by Conley and Wood in 1975 [20]. Phage whiskers have been proposed to function as environmental sensors for salts. These intriguing studies characterized the organization of a single virus as well as a mode of interaction with the host bacterial surface. However, possible consequences for the group behavior (GB) of viral particles and for phage dispersion state were not addressed.

The mechanism and selectivity of sensing the proximity of host bacteria by phage has become an important issue in the field of bacteriophage biology [5]. Yet the contribution of ionic gradients remains largely unknown. Both gram-positive and gram-negative bacteria, including *Escherichia coli*, express cation transporters in the cell membrane, which permit ion conductance that generates [21] high ionic extracellular zones in proximity of outer membrane.

The capability of phage to utilize ionic conditions to sense the microenvironment for induction of survival mechanisms has so far not been addressed. Based on previous work that demonstrated a robust response of phage to the ionic composition at the ion/virion interface we explored the virion/virion interaction regulated by the ionic milieu. Here, we focused on the effect of the alkaline element, sodium.

In addition, we undertook efforts to reduce the complexity of experimental setup by applying a highly efficient purification procedure on phage prior to our experiments; we limited the components of the culture media to the alkaline monovalent cation, sodium, and performed all the experiments in a well-controlled in vitro environment, in isolation from host bacteria.

Under stringent conditions we found a robust effect of sodium on the phage dispersion state. We discovered that lowering ionic strength below a critical threshold triggered a dramatic, high velocity aggregation of phage that appeared to be an all-or-nothing reaction (non-linear), since it simultaneously stimulated all the virions present in the test tube to respond in the same manner, either to aggregate or to disperse, when shifted to low or to high ionic strength environment, respectively. Importantly, the phage retained biological activity while aggregated. Our study shows for the first time, that alkali monovalent cations, Na^+^ and K^+^, act as a critical signal that regulates the bacteriophage state of aggregation.

## Results and discussion

### Aggregation of bacteriophage T4 triggered by low ionic strength media visualized by atomic force microscopy and scanning electron microscopy

Atomic force microscopy (AFM) and scanning electron microscopy (SEM) permits topographical scanning of immobilized nanoparticles. Together, these methods permit complete sample characterization at nanometer resolution [9, 22]. Collectively the techniques allow a reliable estimation of dispersion/aggregation of nano-objects such as those by bacteriophages.

**Fig. 1:**
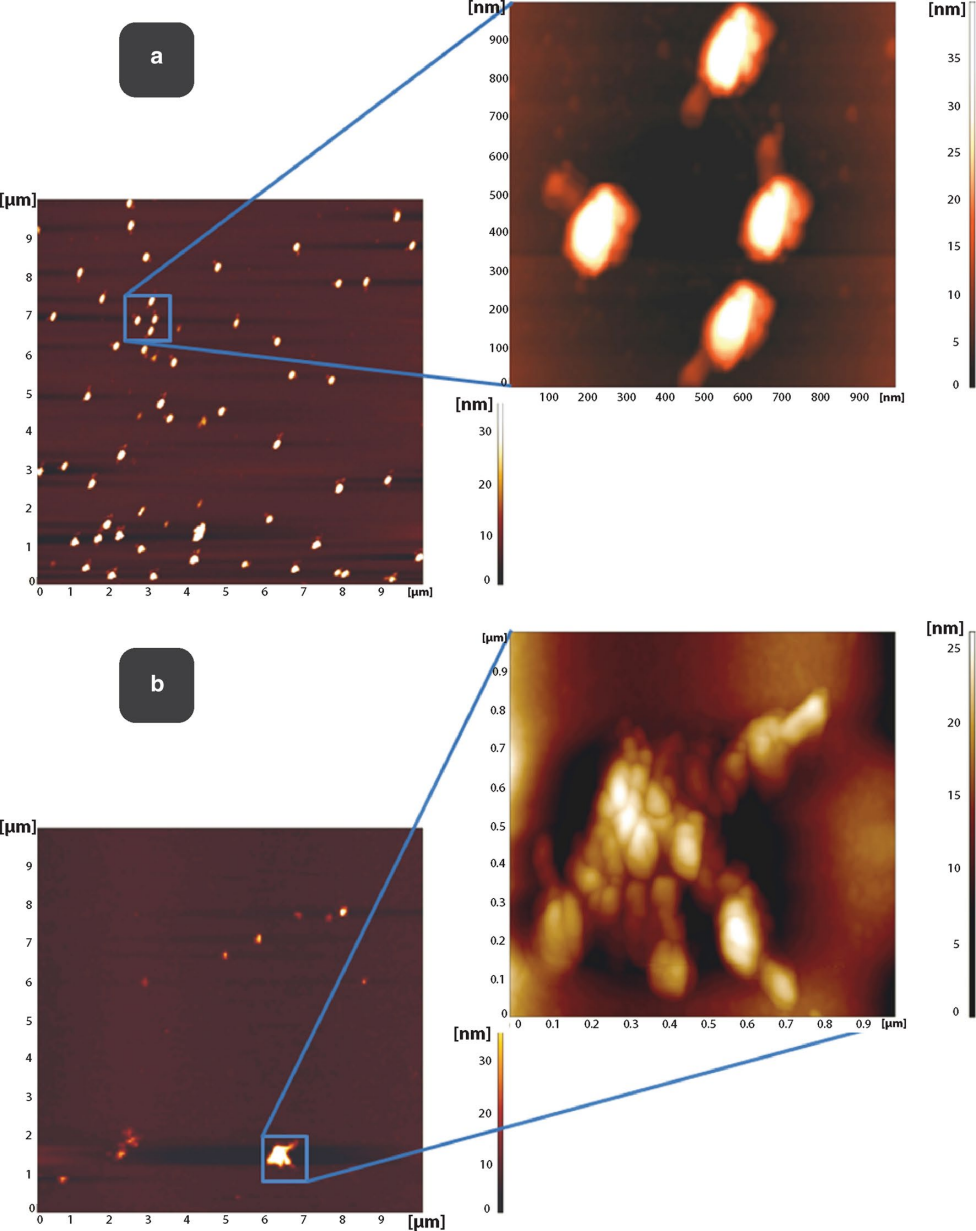
Aggregation of bacteriophage T4 visualized by atomic force microscopy, AFM. **a**. AFM images of T4 bacteriophages on PEI (polyethylene imine) modified mica surface deposited as separate objects from 150 mM NaCl solutions. Scan area 10 × 10 μm. **b**. AFM images of T4 bacteriophages on PEI (polyethylene imine) modified mica surface deposited in clusters from low ionic strength solution (10 mM NaHCO_3_) after 100 min of incubation at room temperature. Scan area 1 × 1 μm

**Fig. 2:**
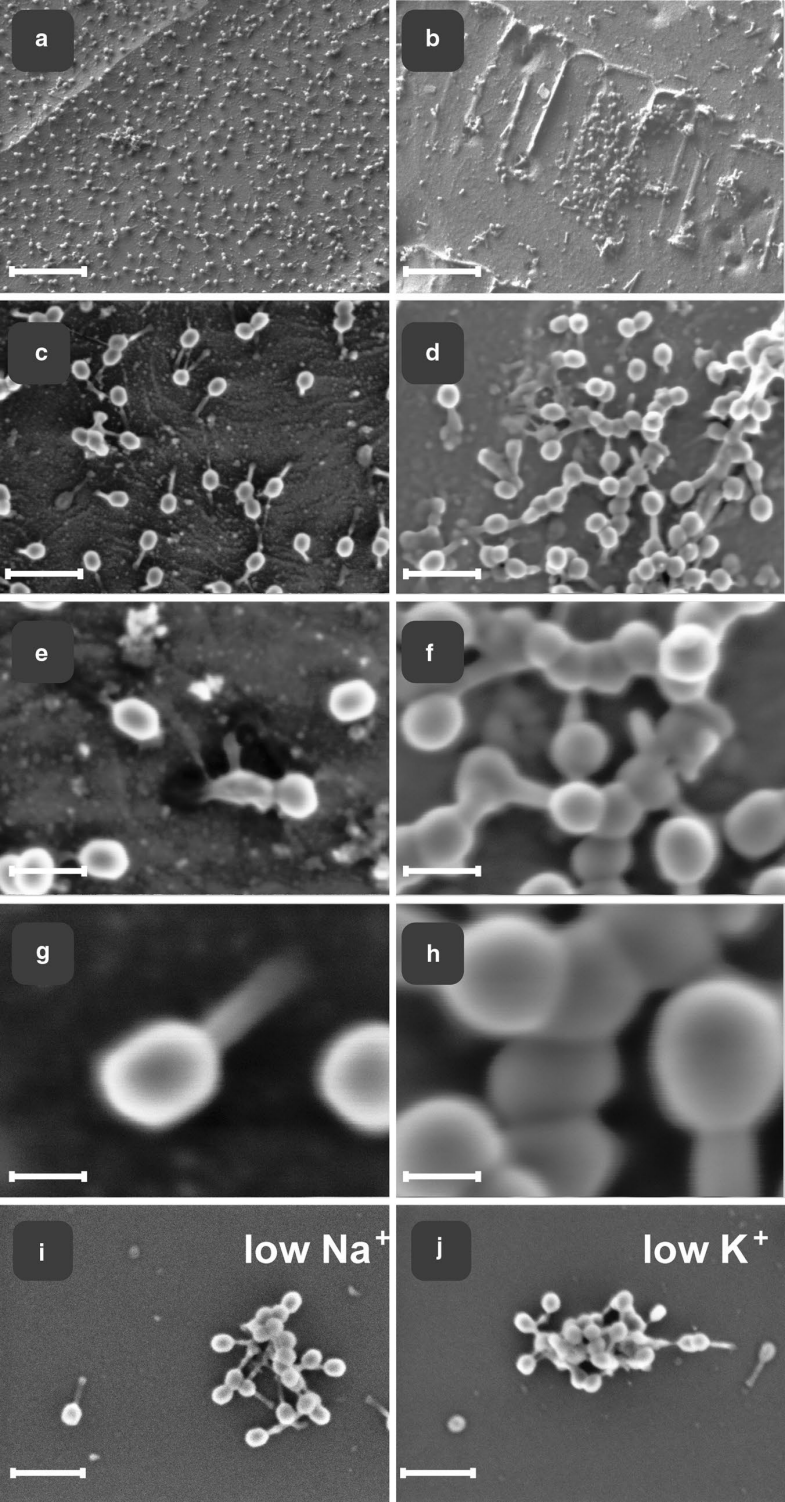
Aggregation of bacteriophage T4 visualized by scanning electron microscopy, SEM. In high-ionic strength 150 mM NaCl bacteriophage particles distributed uniformly on a silicon surface, as separate objects (**a**, **c**, **e**, **g**), while in contrast, in low-ionic strength (10 mM) phage particles get organized in clusters (aggregates) (**b**, **d**, **f**, **h**–**j**). Images represent the typical forms of phage aggregates. Distribution of phage particles depended on solute, namely physiologic 150 mM NaCl (**a**, **c**, **e**, **g**) compared with low ionic strength 10 mM NaHCO_3_ (**b**, **d**, **f**, **h**, **i**). Visible phage particles, deposited on silicon substrate. In-lens SE1 detection (1.2 kV). Note the dispersed phenotype at higher salt concentrations (*left panel*), while aggregation of phages at low salt concentration (*right panel*). **g** Set of representative phage particles at high magnification, with high dispersion, under high (physiologic 150 mM NaCl) solute concentration. SEM scanned at low beam accelerating voltages with SE detection at 1.2 kV acceleration voltage of primary beam. **h** Set of representative phage particles at high magnification, clustered, under low (10 mM NaHCO_3_) solute concentration. In-lens SE1 detection at 1.2 kV acceleration voltage of primary beam. **i**, **j** SEM images of T4 bacteriophages on silicon (100) crystal surface deposited in clusters from low ionic strength solution with cation of sodium as 10 mM NaHCO_3_, (**i**) or with cation of potassium as 10 mM KHCO_3_, (**j**). Please notice a similar morphology of aggregates in both cases, when at low Na^+^ or at low K^+^. *Scale bars* **a**, **b** 1 µm; **c**, **d** 250 nm; **e**, **f** 100 nm; **g**, **h** 50 nm; **i**, **j** 250 nm

T4 particles in 150 mM NaCl were visualized by AFM. The imaging demonstrated that bacteriophage particles were deposited uniformly onto a modified mica surface, as separate objects of dimensions comparable to a single virion (Fig. 1a). In agreement, similarly treated samples prepared for SEM also showed that T4 particles were deposited onto solid silicon substrate in a uniform manner and dispersed as single virions (Fig. 2a). The characteristic shape of intact T4 phage particles was observed under higher magnification using both AFM (Fig. 1a) and SEM (Fig. 2g).

Lowering of the ionic strength of sodium in the phage suspension to 10 mM using NaHCO_3_ resulted in a dramatic clustering of phage particles as visualized by both AFM and SEM (Figs. 1b, 2b, respectively). Under these conditions, the distribution of particles changed to discrete, non-uniform, focal aggregates on a modified mica surface (Fig. 1b, AFM) or on silicon crystal (Fig. 2b, i, SEM). Phenotypically, aggregates were similar in size when visualized by either AFM or SEM (Figs. 1b, 2b, d, f, h, i). Use of potassium, (10 mM KHCO_3_) was also capable of triggering aggregation of T4 phage into clusters similar to those observed with sodium (Fig. 2i, j). Multiple local foci of multi-virion assemblies were generated simultaneously in the whole volume of phage suspension, suggesting a non-linear process of object grouping. These results demonstrate, for the first time, the dramatic and complete aggregation of T4 phage triggered by exposure to a low ionic strength microenvironment.

### Kinetics of bacteriophage aggregation triggered by low ionic strength

**Fig. 3:**
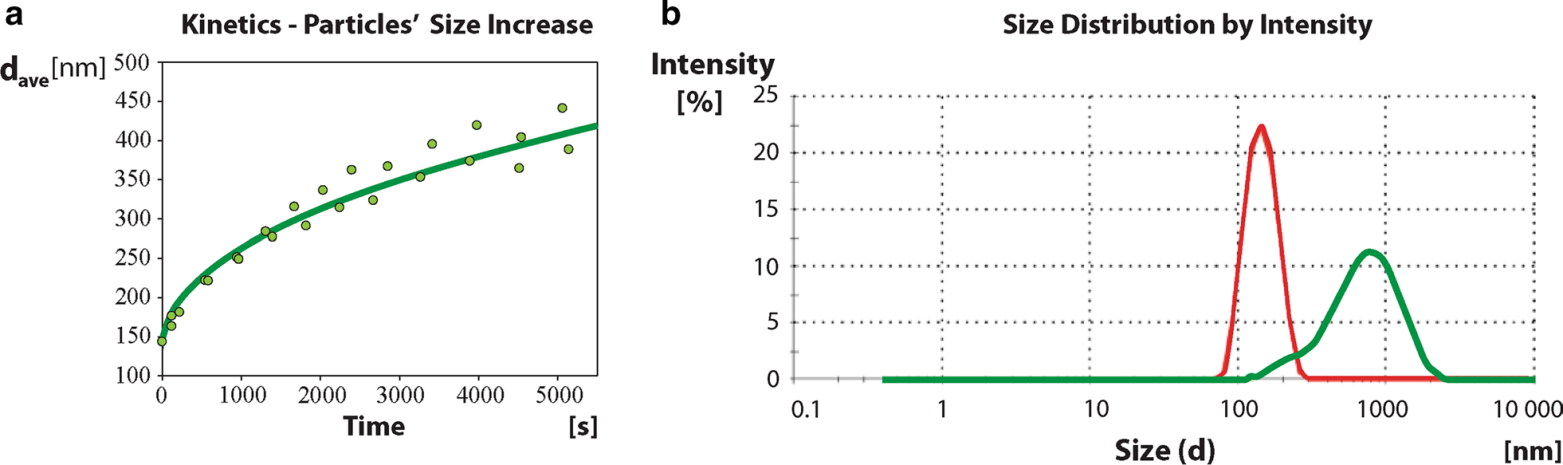
Kinetics of phage aggregation—dependence on ionic strength. **a** Under low ionic strength, the particles of phage rapidly aggregate to form clusters. The initial high rate of aggregation gradually slows down, with plateau in the late phase. Measured values fit to square-root function curve, shown in green. **b** The curves demonstrate DLS analysis where the particles of phage do not cluster when in high ionic concentration (*red curve*) while, upon ionic strength switched to the low range, the aggregation followed (*green curve*). These two contrasting behavior modes correspond to the clustering of nanoparticles, dynamic under liquid suspension conditions, and correlate well with observations of the static methods SEM and AFM

The kinetics of phage aggregation upon exposure to low ionic strength media was measured by determining the hydrodynamic size of phage particles by dynamic light scattering (DLS) as a function of time. In order to simulate native conditions, measurements were made on virus hydrated in fluid media composed of biocompatible elements. The average value of T4 phage diameter in the standard solution (150 mM NaCl, 10 mM NaHCO_3_, 1.2 × 10^11^ [pfu/ml]) was 137.9 ± 1.06 nm and size distribution of the phage particles was narrow, as characterized by the value of the polydispersity index (PDI), 0.030 ± 0.023 (Fig. 3). The results derived from photon-based principles of DLS (Fig. 3) are within the range that corresponds to those obtained in SEM, based on electron-beam principle (Fig. 2). These findings indicate that values determined when phages are dried (SEM) correspond appropriately with values determined when phages exist in a native hydration state (DLS). The diameter was calculated from the measured mean diffusion coefficient equal to 3.40 ± 0.02 μm^2^ s^−1^.

A dramatic change in distribution of T4 particles was observed when ionic strength was lowered to 10 mM. The curve characterizing phage particle behavior when transferred to the low ionic strength environment revealed a rapidly-increasing contribution of larger objects, reflecting the aggregation (grouping) process (Fig. 3a). A rapid progression of aggregation immediately after the change in environmental condition was followed by a slowed rate of aggregation. The mean particle diameter increased approximately with the square root of time, indicating a process controlled by diffusion (Fig. 3a). We noted that the end-result of the process depended on the salt concentration. Therefore, we investigated aggregation after reducing the ionic strength below 20 mM by diluting with 10 mM NaHCO_3_ (Fig. 3b).

Phage aggregation in the low ionic strength solution appeared to be a highly *dynamic* process, causing significant changes in the distribution of individual phage particles, as predicted from AFM and SEM *static* analyses. In this polydisperse system particle clusters differed in size and shape. Therefore, ‘average diameter’ at a particular time point should not be interpreted as the typical size of aggregates. Instead, a general increase of aggregate size over time was characteristic for clusters of various sizes. Polydispersity of phage in solution was typically high. Accordingly, T4 phage in the 10 mM NaHCO_3_ solution used for DLS analysis showed an average effective object diameter of 534 nm and a PDI of 0.32 in solution.

Since the DLS provides values based on a spherically-shaped model, and phage does not completely fit this model, we took advantage of precise measurements of particles by high resolution AFM and ultra-high-resolution SEM, described above. The dimensions of phage clusters found in AFM and SEM confirmed those calculated from DLS and the imaging provided detailed contours of the clusters.

**Fig. 4:**
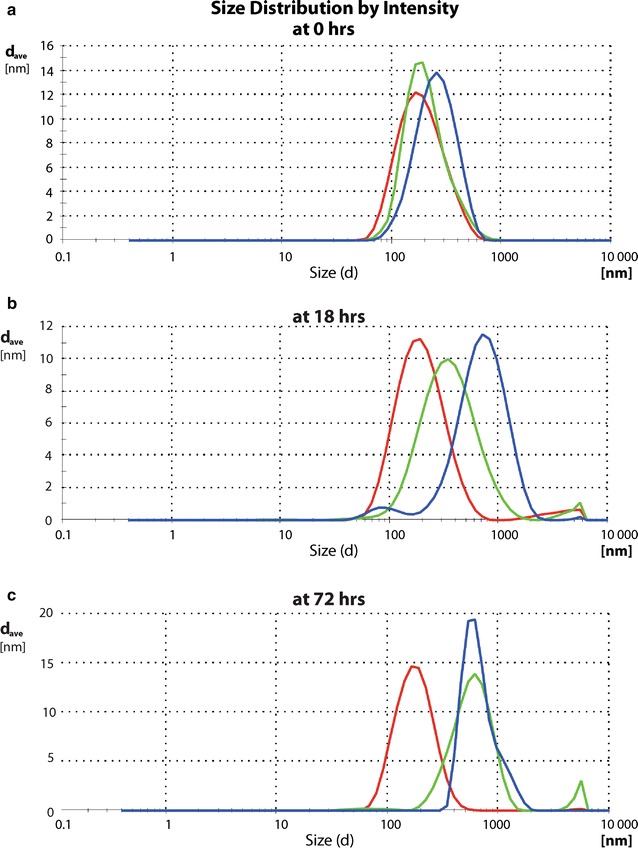
Dependence of aggregation on pH shown at time points **a** 0 h, **b** 18 h, **c** 72 h. Phage aggregation triggered by low ionic strength showed variable dynamics depending on pH. Under lower pH (5.8) we observed the slowest rate of aggregation (*red line*), intermediate rate at neutral pH (7.0) (*green line*) while the fastest under alkaline conditions (pH 8.6) with almost complete contribution of large objects already after 18 h (*blue line*). Aggregation reached the high yield level at the neutral pH only after 72 while at alkaline pH already 18 h were sufficient

10 mM NaHCO_3_ buffer had a pH of 8.64. Therefore all measurements of aggregate size in NaHCO_3_ were performed in freshly prepared solutions at this slightly alkaline pH. To determine whether pH affected the aggregation process, and to assess the role of HCO_3_^−^ anions, we used H_2_PO_4_^−^ and HPO_4_^2−^, rather than Cl^−^ to regulate pH of the media; the samples in phosphate buffer of I = 0.01 and pH of 5.80, 7.00 or 8.64 were prepared. Aggregation was not observed at the slightly acidic pH of 5.8 (Fig. 4a–c, red curve), but was observed at neutral (green curve) and alkaline pH (blue curve). These results suggest that aggregation is not dependent on the isoelectric point of a whole T4 virion (pI = 4) [23]. Aggregates formed in phosphate-containing solutions were of similar size to those measured in 10 mM NaHCO_3_ (I = 0.01). However, in neutral pH, the aggregates stabilized at a slower rate than those formed under slightly alkaline conditions (Fig. 4a–c).

**Fig. 5:**
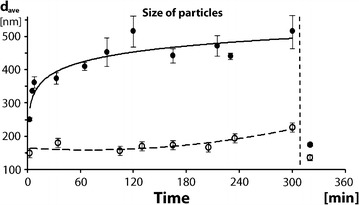
Aggregation of phage in dependence on temperature. Inhibition of aggregation at low temperature. Average effective particle diameter of bacteriophages in 37 °C (*full points*) in comparison to 4 °C (*empty circles*). The curve of a square-root function–the best fit for data measured at 37 °C—suggests a diffusion process being involved in the phage-aggregation progression. Dashed curve, fitting the measured particle dimensions under an inhibitory temperature, shows low starting value of particles’ size and much slower increase throughout timescale of the experiment (*empty circles*). *Vertical line* after time point ‘300 min’, indicates the addition of concentrated salt to previously formed aggregates to test reversibility of the aggregated state, triggered by high ionic strength. Please note a dramatic drop of average particle size at high salt (*right part of the image*, between time-points 300 and 360 min). Dispersion was noticed not only population of phage aggregated at 37 °C, but also in the less aggregated population at 4 °C, speaking for dispersion of clusters into individual phage particles in high ionic strength thus demonstrated the reversibility of the whole process, at both temperatures

The dynamics of phage aggregation depended strongly on temperature. At 37 °C, the aggregation occurred almost instantly after ionic strength was reduced. Phage continued to aggregate with time (Fig. 5), reaching an average cluster diameter of 470 nm by 90 min, after which aggregate size stabilized with only minor fluctuation. At 4 °C, aggregation was substantially reduced with average aggregate diameters of less than 250 nm, even after 300 min. Importantly, aggregation could be promptly stopped and reversed by restoration of high ionic strength (Fig. 5). These results provide evidence that low-salt triggered aggregation of T4 phage is a reversible process.

### Retention of phage aggregates on micro-filters

Bacteriophage aggregates are substantially larger than non-aggregated phage particles, thus we compared retention of aggregated and non-aggregated phage particles on standard micro-filters (0.22 µm), expecting that a larger proportion of aggregated forms would be captured. Indeed, phage recovery was 2.5-times higher (p < 0.0012) when solutions containing the aggregated phage particles were passed through the filters compared to solutions containing the dispersed phage: bacteriophage in 150 mM NaCl (mean ± SD = 4.0 × 10e8 (±1.8) [PFU/ml], n = 6) versus 10 mM NaHCO_3_ (mean ± SD = 10.4 × 10e8 (±2.6) [PFU/ml], n = 6). These findings demonstrate that aggregated bacteriophage can be efficiently collected with standard micro-filters.

### The effect of phage aggregation on its biological activity

To determine whether bacteriophage aggregation induced by physical conditions has biological consequences on phage viability or bioavailability, we compared the activity of T4 phage infection in *E. coli* strain B (expressed as pfu ml^−1^: plaque forming units per ml) in standard culture media and low-salt media. We chose bacteriophage T4, a tailed virus (*Caudovirales*, *Myoviridae*), since it is a well-established model of bacteriophage with a wide application [24–27]. Importantly, its host, *E. coli*, is an important marker of human-born water contamination. The number of plaques formed by T4 phage in low-salt media was about half of that obtained under standard culture conditions: when bacteriophage was cultured in normal (high ionic strength) 150 mM NaCl, the mean number value (±SD) was 2.9 × 10e8 (±0.6) [PFU/ml, n = 6] compared to 1.4 × 10e8 (±0.18) [PFU/ml, n = 6], when ionic strength was decreased by dilution with 10 mM NaHCO_3_. Taking into account the geometric rate of growth in phage, this result, when expressed as plaque-forming activity, demonstrates that bacteriophage aggregation exerts a moderate inhibitory effect on phage infectivity. The inhibitory effect of aggregation on infectivity could be successfully reversed by dispersion of aggregated phage upon return to higher ionic strength media, as shown in Fig. 5. This implies reversibility of the aggregation process.

**Fig. 6:**
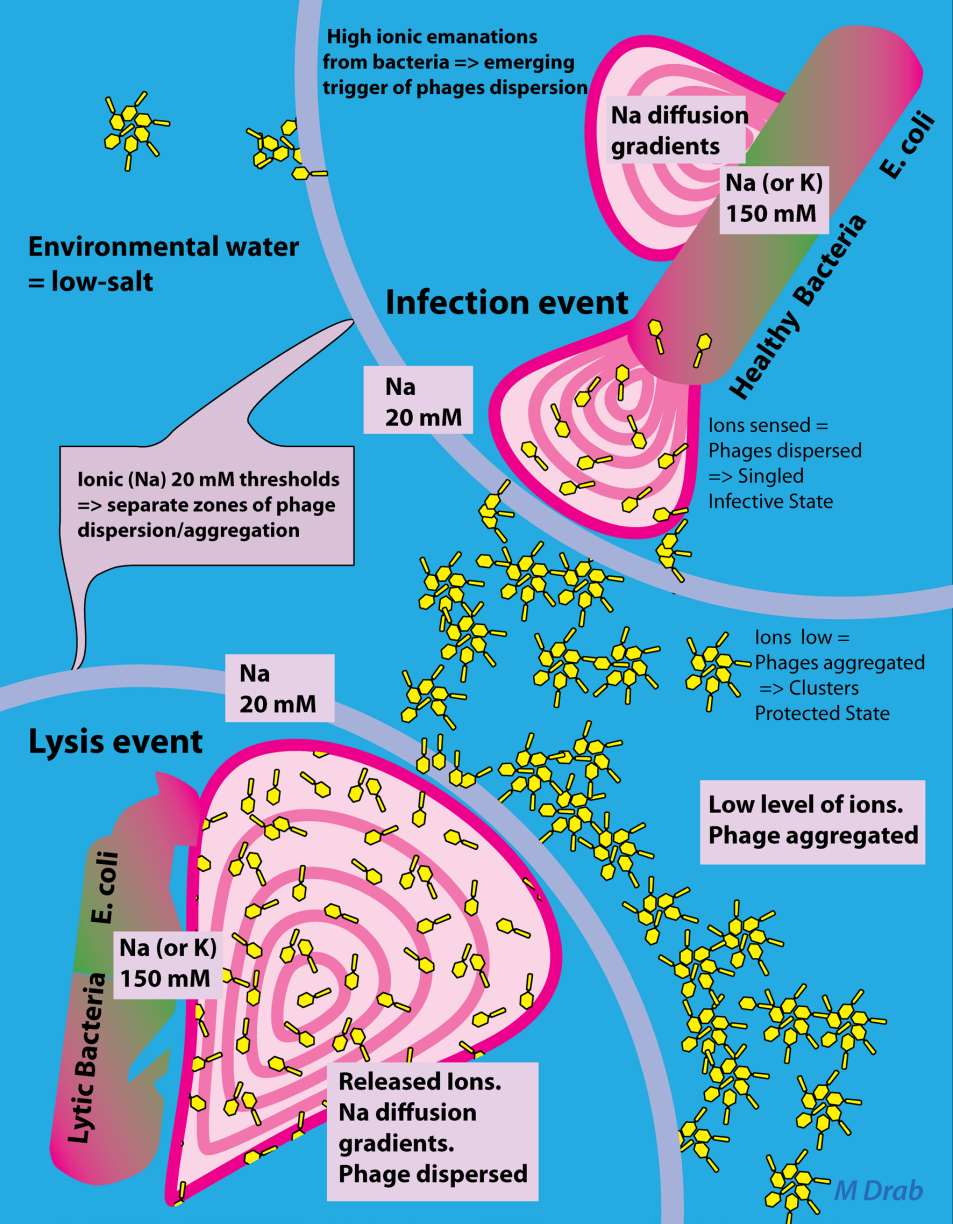
Ion availability as a trigger for aggregation/dispersion of bacteriophage T4—proposed mechanism in phage survival/infectivity cycles in (micro)-environment—the locally increased ions in proximity of bacteria serve as a cue, sensed by the phage, and converts into a quorum signal for re-organization. Infected (lytic phase) bacterium concomitantly releases cytosolic ions at high concentrations and phage in its dispersed form (*left lower panel*). Upon crossing the threshold gradient (range of 20 mM Na^+^, depicted as *magenta circles*) of monovalent cation, in low-ionic strength (water in soil) phage particles get clustered (aggregates), presumably to prolong bacteriophage survival (*middle image panel*). In contrast, in higher ionic concentration, like when immersed in zone of ionic fluxes contributed by live bacteria, bacteriophage particles disperse from clusters, as separate objects, to broaden the spread area and increase invasion events in proximity of the sensed host (*right upper panel*)

Our study demonstrates for the first time that the ionic strength of the fluid environment serves as a pivotal mechanism to determine aggregation (grouping) and dispersion (ungrouping) of T4 bacteriophage particles. We describe a dramatic, yet reversible, process of T4 phage aggregation that is dependent on ionic strength in microenvironment as schematically illustrated in Fig. 6.

Cation concentration effectively controlled the aggregation/dispersion state over a broad range of pH and temperatures, while varying anions did not affect the capability of sodium to regulate the aggregation/dispersion state. However, pH was an important modifier of the kinetics of aggregation; slightly alkaline solution accelerated the aggregation, when compared to neutral pH media. Notably, the size of clusters in alkaline and neutral pH solutions stabilized at the same diameter when sufficient time was allowed to complete the reaction (Fig. 4). In contrast, an acidic solution with an iso-electric point similar to that of T4 phage did not permit aggregation of the virus.

Our data reveal a new mechanism of bacteriophage regulation that is based on the ionic strength of the alkaline monovalent sodium cation, Na^+^. The state of phage aggregation advances knowledge of the phage ecology, life cycle, in the context of virus classification as reported by others [10, 16, 18, 24]. We propose that low-cation triggered phage aggregation reflects a new mechanism in an evolutionary adaptation to environment [16].

Prior studies frequently reported on the factors that complicate the study of phage, such as poorly defined media, enzymatic impurities, multi- and off-target effects of divalent cations, as well as the complex communication network between phage and its host. Therefore, we decided to simplify and fully define our experimental conditions. In order to improve control of phage in our experimental paradigm, we used an in vitro approach that contained only T4 virions, without its host bacterium *E. coli* context. We also used highly defined media that were restricted to the single monovalent sodium cation, thus avoiding the enzymatic effects controlled by the bivalent cations, magnesium and calcium. We also purified the phage with our patented method that remove bacterial impurities, (i.e., lipopolysaccharides of host bacterium) and thus further substantiates applicability of our work for phage used for human therapy [28].

Our study differs substantially from previous work that addressed aggregation as a biological mechanism. The most important differences were: the simplicity of the factors and media used, highly-purified phage, isolation from host bacteria, and exposure to a single monovalent metal cation. The range of ionic strength examined from 10 mM up to 150 mM, remained entirely within the typical ionic strength of the phage bio habitat. In prior publications by others, much higher ionic concentrations were used which evoked osmotic shock in phage. Though we tested “high-ionic strength” solutions, sodium concentrations were always within levels habitable for most living cells, (150 mM). In order to determine whether the observed phenomenon was related to sodium specifically or to positive charge of monovalent alkali cation in general, we used another biologically-relevant cation, potassium, as 10 mM KHCO_3_ salt. We compared the effect of sodium and potassium ions on phage biological activity, which appeared strikingly similar in both cases (2.3 × 10^11^ PFU/ml in 10 mM Na^+^; 2.5 × 10^11^ PFU/ml in 10 mM K^+^). Finding of the similar biological activity and formation of morphologically identical phage aggregates (Fig. 2i, j) prompted us to conclude, that both monovalent cations, Na^+^ and K^+^, exert comparable effects on the phage aggregation state and support the same biological activity.

Serwer and co-workers performed pioneering work on phage aggregation. Using typical phage cultures, containing host bacteria, these investigators observed aggregation in natural phage plaques with single-molecule fluorescence microscopy [26]. Their work unambiguously defined the biological importance of phage aggregation [14], in its natural life-cycle. According to the work of Serwer et al. phage aggregates lowered the propagation potential of lytic bacteriophages but, at the same time, aggregation appeared to assist phage stabilization under harsh conditions [14, 16]. Serwer suggested that aggregation was part of an evolutionary trade-off that increased the rate at which phage could find its host [14]. Aggregation may also provide a means by which lytic bacteriophages optimize their reproduction [18].

*Escherichia coli* bacteria express cation transporters such as Na^+^/K^+^ anti-porters in their membrane, which permit ion flux [21, 29] and generate zones of high-ionic strength close to the membrane. Similar cation transporters have been found in other bacteria, including human pathogens [30]. Activity of these, mostly high-yield anti-porters, locally enrich the microenvironment with ions; thus a discrete ionic zone formed on the bacterial surface creates a distinct environment that differs from the surrounding soil or water.

In our proposed mechanism, low salt conditions operate as the key trigger for viral aggregation, which becomes a natural course of the bacteriophage life cycle.

In our model, when phage leave the host bacteria and enter the low-salt environment, phage virions aggregate in a GB mode, thus enabling survival until they reach the next host. The model of aggregation/disaggregation cycles is depicted in Fig. 6. From the left lower quadrant, phage start in high-ionic environment (~150 mM) made up of alkaline monovalent cations. In our model, the high-ionic strength media represents the properties of the phage host cytosol. The lysis of bacterial cells completes the infection course of phage. With the release of the host cytosolic content, including monovalent cations, sodium and potassium, phage diffuses much more slowly than do small ions, as a result of substantial molecular weight difference between a single cation and a whole virion. Slow wave of diffusing phage, when still in its dispersed form as singled particles, readily passes through the rapidly dropping concentration gradients of cations until dilution of ions reaches a critical threshold value of 20 mM. Once the environmental ionic strength is reduced to 20 mM threshold zone, aggregation is triggered. The aggregation characteristics, defined in the present study, demonstrate that virion clustering operates efficiently, with diffusion velocity being the major limiting factor. The steep slope of the initial aggregation and completeness of the reaction suggests that there is a cooperative process between phage. The small inter-virion distances observed upon aggregation suggest an advantage of cooperative process that operates in the initial phase of phage aggregation. We base our conclusions on a few reliable factors: cytosol volume of single lysed bacterium—as a source of high ionic strength, rapid diffusion of sodium/potassium cations when compared to slow diffusion of phage, and low ionic strength of environmental aquatic habitat of *E. coli*. Under such conditions, we calculated that 150 mM cation becomes diluted to threshold value of 20 mM when bacterium cytosol gets diluted approximately 8-times its volume (for pure environmental water), it is in less than 1.2 µm distance away from bacteria surface. This surprisingly small distance enables phage to organize into aggregates when still near host bacterium, thus virions remain in proximity to one another. Each lysed bacterium yields 100–200 T4 phage particles. Thus, phage probably gets aggregated upon its release from lysed host bacterium, when the chance of sensing, and interacting with another virion is very high. The aggregations of phage contain on average, as we observed in in vitro experiments, 20–100 virions. Aggregates of phage diffuse as entities, remain clustered, and persist stable for hours and days. Upon a rise of ionic strength to 150 mM NaCl, aggregates rapidly destabilize and phage disperses into single virions.

The change of Na^+^ cation concentration causes transition of phages from colloidal liquid state to colloidal locally condensed state, reminiscent of a focally-appearing sol-to-gel transition. This signal is similar to some extend to the Ca^2+^ signaling in Eukaryotes which causes sol-to-gel transitions of cytoplasmic components [31]. Actin polymerization induced by a free intracellular ionic calcium rise ([Ca^2+^]_i_) causes (in Eukaryotes) whole-cell cytoskeletal contraction. The rapid sol-to-gel transition prevents rupture of the cell as an entity and the cell’s mechanical properties make it more resistant to strain and stress. The analogy of sol-to-gel transition between calcium cation-induced in Eukaryotes and sodium cation–induced in phages is further supported by the reversibility of both; reverting of cation’s level to the resting cytosolic value causes reciprocal, gel-to-sol transition in both, the cell cytoskeleton and phage. Our findings on sodium effects in phages aggregation (sol-to-gel transition type of reaction) suggest Na^+^ may serve as a signal, similar to some extent to the role played by [Ca^2+^]_i_ in higher organisms. Hence, we propose Na^+^ is a triggering signal in phages, enabling QS and GB.

The aggregated-to-disaggregated transition observed in vitro in the present study, mimics the condition when a package of clustered virions approaches another bacterial host cell (Fig. 6, right upper quadrant). Ionic fluxes are an inherent property of living *E. coli* and other bacteria, due to the expression of cation-exchangers in their membranes. Ionic fluxes from bacteria are sensed by the aggregated phages and the process of disaggregation follows, in agreement with the ionic threshold reached again, in increasing cation gradient, reversal of initial lysis event. Dispersed, single virions have greater chance of infecting a new host than the whole aggregate, since single particles rotate freely and as such they more easily approach the host surface at a permissive angle and establish the necessary tight contact with their tail plate, as well as their long and short whiskers. Strategically, the advantage of such cycles of aggregation/disaggregation would be cooperative infectivity, since bacteria often exist in groups, for example as dividing population, a colony or biofilm. The detected presence of bacterium, sensed by phages in our model as high ionic fluxes from membrane anti-porters, increases the chance for multiple infections from the same phages package. In addition, as previous works demonstrated, phages in aggregates are more resistant to harsh conditions of environment, such as heat.

Our model, based on robust data of in vitro experiments, operates with high fidelity under well-controlled conditions of our experimental workflow. Proposed hypothetical projection of the in vitro tested model into the real bacteria-phage infection is to be studied in detail in further work. However, using SEM, we observed that lytic bacterial ghosts contain phages adhered on their surface in both single and clustered form, in agreement with proposed fluctuations of ionic gradients upon cytosol release (data not shown) and in support of our simplistic concept.

These ion fluxes from bacteria [29, 30] (in Fig. 6 collectively named “ionic emanations”) may serve as a signal for phage [30, 32] and as such prompted us to formulate *evolutionary* and *environmental* projections from our in vitro-tested model. The ionic fluxes would serve as a physiologically important cue that, by dispersing phage virions, enable again an individual virion to interact with bacterial wall. The cycles of aggregation–dispersion would be a novel phenomenon, that evolved based on ionic strength differences between intra- and extracellular milieu by host bacteria—mild versus harsh conditions for phage. An interesting ecological aspect emerges within a broader perspective of our study. Since mineral and bioorganic fertilizers from agricultural activities contain ionic compounds, they may influence the phage survival in modern modifications of soil worldwide. Due to effects on the microenvironment, they could potentially act on macro-scale ecology.

Kinetics of the transitions process between states of aggregation/dispersion was another interesting aspect of our study. Phages aggregation was instantly induced by transferring the phages from 150 mM Na^+^ to 10 mM Na^+^ but the clustering could be promptly stopped and reversed by restoring the physiological ionic strength of 150 mM Na^+^. We calculated the threshold ionic strength to be in the range of 20 mM Na^+^, when aggregation was measured at optimal pH of 8.64. Selection of solutions’ composition was based on the known fact that HCO_3_^−^ anion plays a significant role in stabilizing the pH of the living organisms across an evolutionary tree, from archaea to humans [33, 34]. The range of aggregation in low ionic strength medium correlated with time of incubation while its kinetics depended both on the temperature (Fig. 5) and on pH (Fig. 4).

Our work demonstrates a novel phenomenon in phage behavior. In previous work on phages (RNA viruses) [35] it has been observed that process of phages aggregation was induced by pH value close to pI of the virion, the loss of electrostatic repelling could explain this behavior. In our case, the aggregation was triggered at neutral or slightly alkaline pH, at pH far away from pI of T4 phage (pI = 4 for T4 phage) [24]. Moreover, aggregation was *inhibited* in acidic range of pH, where the pI is expected. Therefore, the loss of electrostatic repelling cannot explain the aggregation of T4 virions. Instead, strong attractive forces are triggered by loss of ionic strength in our experimental setup. The attraction mechanism appears to be an active process capable of rapid virion clustering. This type of active attraction seems to substantially differ from previously published aggregation processes at pI, which is passive, primarily driven by diffusion; in our case the diffusion is the limiting factor. The onset of aggregation in the first minutes resulted in steep increase of kinetics size curve that continued its rise until availability of single virions declined. As a result, the curve flattened to reach a plateau (Figs. 3, 4, 5), in a typical exhaustion-of-substrate reaction, with phage playing the role of substrate. The differentiation of the contribution of pH, Na^+^ cation and HCO_3_^−^ anion in clustering was performed in experiments presented in Figs. 3, 4 and 5. The contribution of sodium appeared to be the crucial factor for aggregation, since it occurred both, in the presence of HCO_3_^−^ anion (Figs. 3, 5), or in its absence (Fig. 4, here anion HCO_3_^−^ was replaced by H_2_PO_4_^−^ and HPO_4_^2−^ anions). In all those scenarios, the sodium cation Na^+^ was a sufficient factor capable of triggering aggregation (when low) or dispersion (when high) of phages aggregation, while HCO_3_^−^ could be replaced with H_2_PO_4_^−^/HPO_4_^2−^, provided pH was set at adequate values (neutral to slightly alkaline).

Importantly, Lark and Adams have raised aspects of phages aggregation in 1953, when the authors explored the heat-resistance of phage [11]. Their studies focused on identifying factors that prevented killing of the phages under hyperthermia. Alkaline cations appeared, based on their studies, as key protecting factor, whose concentration directly and positively affected the survival rate. Both monovalent and divalent ions exerted sensitizing effects, with large effects by sodium, potassium and magnesium, but not calcium. Physical chemistry of reaction, with phage and cations as its substrates, allowed identification of cation-binding sites in phage virions and pointed to the key importance of phage-cation interactions for phage biology. These fundamental and profound studies allowed appreciation of salt concentration as a predictable and robust regulator of phage survival under high temperatures. However, during these elegant physicochemical studies, the conditions used were extremely harsh, in accordance to the major aim of their authors: how to efficiently kill the virus. The salt concentrations were used in submolar and molar range and the gradients were generated to rapidly disassemble and kill the phage virions. Authors could not make conclusions about the mechanism of cation effects on phages, largely due to use of complex broth and non-defined media and limitations in available biotechnological toolbox at that time. Importantly, these studies focused on killing the phages rather than studying its survival and phages aggregation was an off-target effect that could be associated with DNA loss from the virion; the aggregation as a possible mechanism of increasing phage survival at mild temperatures was not directly addressed or proposed. In those studies, authors did not address the role of salts or cations at concentrations of living organisms or aquatic reservoirs. The alkaline cation concentration in the 10 mM range was identified by these authors as harmful for phage. However a body of evidence was generated in the context of heat-sensitivity and thermal-killing [11] (different from our phage-survival promoting design).

Phages aggregation can be used to develop methods for virus biotechnological preparations. Aggregated phage virions could be efficiently filtered from low-ionic strength solutions by standard micro-filters and then recovered from filter membranes (Fig. 4). Employing our finding of aggregation/dispersion-triggered by passing the ionic thresholds—may become useful in technological processes that utilize bacteriophages as nano-carriers, biosensors or molecular platforms for drugs and other active substances. Low ionic strength holds promise for processes that benefit from aggregation. Our approach is both cost-effective and robust method of phages aggregation/dispersion, and as such it may improve processes of phage separation from various mixtures, purification, deposition and others, offering new technological solutions.

Quorum sensing (QS) is a signaling system among bacteria that senses secreted molecules and the bacteria adopt phenotypes to react accordingly. There are two mechanisms of quorum sensing in bacteria based on chemical signaling molecule: lactone (Lux) and peptide one [36]. Phages have been implicated in inter-bacterial QS [5, 36, 37]. Phages modify inter-bacterial QS by influencing response regulators. Moreover, they encode the response regulator themselves [36, 37].

To date, QS has not been described in viruses. Our data implicate the ionic composition in microenvironment as a chemical signal that enables QS in phage T4, for the first time. If the definition of QS can be transferred from bacteria to virus, then our findings in T4 phage obviously meet the criteria set for bacteria: chemical signal and phenotypic response of the population of organisms to this signal [36].

An intriguing question emerges: How does this highly efficient mechanism operate to drive aggregation/disaggregation? Triggered by ionic strength fluctuations high-to-low, aggregation seems to be driven by mechanism depending on electrostatic switch. However, if we take into account the dramatic difference between kinetics at 37 and 4 °C, aggregation cannot solely be explained by dipole charge coupling. Our observation that phage aggregation was dependent on pH, with maximum of aggregation rate reached at slightly alkaline pH, far from isoelectric point, provides direct evidence that electrostatic repellence is not sufficient to overcome strong virion-virion attracting forces (Fig. 5, right part). The best aggregation effects noticed at pH far from pI of T4 suggest other, perhaps structural, strong clustering forces. Phage whiskers are a probable candidate given prior evidence that they are involved in sensing ionic strength in the surrounding environment [20]. Therefore, structural changes seem to act as a consequence of cationic interaction with phage. Whatever the operating mechanism, conceptually, the group reaction of phage virions on such a clear simple signal as sodium ion concentration in an all-or-nothing manner allows us to propose the phenomenon of quorum sensing in phages. T4 bacteriophage, as an obligatory parasite, seemed up to now not to possess the machinery of signaling. However, our findings suggest that sodium cation derived from the environment serves as a simple and robust signal for phage/phage sensing. Here, we propose for the first time that QS and GB are exhibited by viruses.

## Conclusions

Our study shows, for the first time, that alkaline monovalent cations Na^+^ and K^+^ act as a critical signal that regulates the bacteriophages state of aggregation. Aggregation of phage particles triggered by low ionic strength is a newly identified mechanism of viral regulation.

Our data raise the possibility that group behavior and quorum sensing operates in viruses. The hypothesis of QS in viruses that has emerged from our studies may open an interesting direction of study in the future.

The mechanism of aggregation promises multiple benefits in the areas of bacteriophage-based biotechnology, medicine, basic science, as well as evolutionary and environmental microbiology [9], where the identification of a physical switch to invoke clustering of bacteriophage particles might be beneficial. The reversal of aggregation without toxicity to virus promises breakthrough in preparative biotechnology of phage-involving processes since it enables retention of phages in their viable form on standard micro-filters.

## Methods

### Chemicals

The agar and tryptone were purchased from Biocorp, Warsaw, Poland. McConkey agar and beef extract were purchased from BTL, Lodz, Poland. Glucose was purchased from Baxter, Lublin, Poland. All other reagents and solvents were purchased from Sigma-Aldrich, St Luis, USA.

### Bacterial strains

*Escherichia coli* B strain was obtained from the Polish Collection of Microorganisms at the Institute of Immunology and Experimental Therapy (IIET), Polish Academy of Sciences, (PMC 1630). Standard bacterial cultures were maintained on McConkey agar at 4 °C and subcultured on the same agar during the course of experimentation.

### Bacteriophage preparation

Bacteriophage T4 was obtained from the American Type Culture Collection (Rockville, Maryland, USA). The strain was stored in the Polish Collection of Microorganisms at IIET, Polish Academy of Sciences. To propagate the phage, poor medium based on casein acid hydrolysate was applied, as established [28]. *E. coli* B was cultured in SM-1 medium (1.0% (m/v) acid casein hydrolysate (Hy-Case Amino), 0.35% (m/v) K_2_HPO_4_, 0.5% (m/v) Na_2_SO_4_, 1% glucose). The infection ratio of bacteriophage particles to number of bacteria was 1:10, as described by us in [38]. The preparation of bacteriophage lysate was performed in a bioreactor (BIOFLO 415, New Brunswick, USA). The titer of phage particles, expressed as plaque forming units (pfu), was determined using the double layer agar (DLA) technique [38].

### Purification of bacteriophage lysate

In order to achieve high purity of phage, and eliminate the bacterial impurities, particularly LPS, we have applied our patented and published method that was originally developed for human therapy purposes; the purity of phage achieved with our method belongs to the highest currently available standards worldwide [28]. Briefly, bacteriophage lysate was purified by filtration through polysulfone membrane filters 0.22 µm (Merck Millipore, Billerica, MA, USA), then concentrated through Pellicone membrane (1000 kDa) (Millipore, Warsaw, Poland). Afterwards, phage preparations were filtrated on Sepharose 4B and then subjected to heterophase extraction to 1-octanol with 3:4 (v/v) ratio of organic matter to lysate [28]. After extraction, phase separation was performed at 4 °C. The collected aqueous phases were dialyzed against 25% aqueous ethanol solution, and subsequently the sample was dialyzed against 0.15 M NaCl (SERVA dialysis tubing MWCO 12,000–14,000, SERVA Electrophoresis GmbH, Heidelberg, Germany). Finally, the bacteriophage lysate was passed through Pellicone membrane (1000 kDa) (Millipore, Warsaw, Poland). Final preparations were approx. 1 × 10^11^ pfu ml^−1^. These preparations were used for all further experiments.

### Atomic force microscopy (AFM) imaging

Samples for AFM were prepared from a bacteriophage T4 suspension (in 0.15 M NaCl or in 0.01 M NaHCO_3_) by placing 10 μl of the suspension onto the surface of mica modified by a pre-adsorbed layer of PEI (polyethylene imine of M ~70,000 Da). After 15 min of deposition, mica was carefully rinsed with distilled water to avoid solute crystallization.

Such a prepared sample was positioned on the holder for AFM scanning. Sizes and morphologies of phage particles were investigated by AFM imaging in air, using the NT-MDT device with the SMENA SFC050L scanning head and piezo scanning stage. X, Y directions were scanned by a piezo stage and Z direction was measured by head. All measurements were performed in the semi-contact mode by using high-resolution silicon probes (NT-MDT).

### Scanning electron microscopy (SEM)

Scanning electron microscopy of bacteriophage particles was performed at low accelerating voltage of the primary beam without any coating of the samples, as described in previous work [9, 39]. The viral particle suspensions, at concentrations of 1.25 × 10^11^ ml^−1^, were applied onto silicon chips and allowed to adhere at 4 °C for 24 h. The samples were fixed with 2.5% glutaraldehyde in 0.1 M cacodylate buffer for 30 min at 4 °C, then washed in water and dehydrated in series of methanol solutions (25–50–75–100–100%) in 1-h steps at 4 °C. Samples underwent critical point drying with methanol exchanged for liquid CO_2_ in an automatized approach, (CPD300 AUTO, Leica Microsystems, Austria) and imaged with cross-beam scanning electron microscope equipped with Schottky field-emission cathode (Auriga 60, Carl Zeiss, Oberkochen, Germany) at 1.2 kV accelerating voltage, thus the imaging was performed within a mode referred to as the low-voltage, field-emission scanning electron microscopy (LV-FESEM) of non-labeled, critical point-dried sample. Images represent the Everhart–Thornley or in-lens SE detection directly from the sample surfaces, with no coating or contrasting applied [39].

### Size measurement of bacteriophage particles with dynamic light scattering

Particle sizes were determined by DLS using the Zetasizer NanoZS apparatus, Malvern Instruments Ltd., Malvern, UK. This technique is based on the measurement of fluctuations of intensity of light scattered by particles. Since Brownian motion of the particles cause these fluctuations, the registered optical signal is related to the particle diffusion coefficients which can be converted to the size of spherical particles moving with the same diffusion coefficient as the studied object. Sizes calculated this way should be treated as hydrodynamic particle radii rather than so-called hard-core cross-sections. For simplicity, the particle shape effect was ignored.

In order to compare phage aggregation at 37 °C and at 4 °C, a T4 preparation (3.35 × 10^10^ pfu ml^−1^) in 150 mM NaCl was diluted 25 times with 10 mM NaHCO_3_ to promote phage aggregation. The measuring cuvettes were put to 4 or 37 °C. Size distribution was measured as a function of time (5 h) at the designated temperatures. Then 1650 mM NaCl was added to samples (1:12 vol/vol) to restore the initial ionic strength of 150 mM and the size distribution was tested (Fig. 5). To determine the effect of pH on aggregation, three separate phosphate buffers of I = 0.01 (“I” meaning ionic strength), with pH equal 5.80, 7.00 or 8.64, were prepared with NaH_2_PO_4_ and Na_2_HPO_4_. Phage T4 particles (4.9 × 10^10^), initially suspended in 150 mM NaCl, were diluted 50 times with appropriate buffer to start the aggregation process while control samples were diluted with 150 mM NaCl. Samples were measured directly after dilution, then left at room temperature overnight and measured again after 18 and 72 h.

### Phage retention on standard micro-filters

T4 phage was transferred to 150 mM NaCl or 10 mM NaHCO_3_ (0.6 ml, 1.4 × 10^9^ pfu ml^−1^) and incubated at 37 °C for 3 h. Then solutions were passed through a sterile low-protein binding syringe filter 0.22 μm ø 15 mm. Subsequently, the filters were washed with 0.4 ml 150 mM NaCl to recover phages. Titer of each filtrate sample was determined by biological method, as plaque forming units (pfu) per microliter of solution.

### The effect of phages aggregation on their biological activity

To determine biological effect of aggregation, the phage stock preparations (aliquots of 600 μl of 1.4 × 10^9^ pfu ml^−1^) were dialyzed against 150 mM NaCl, 10 mM NaHCO_3_ or 10 mM KHCO_3_ for 24 h.

For comparison of (i) physiological and (ii) low ionic strength conditions, the host bacteria were cultured on two types of solid media, respectively, per liter: (i) Na_2_HPO_4_ 3 g; Tryptone 10 g; Beef Extract 3 g; NaCl 5 g; agar 12 g per liter, (ii) Na_2_HPO_4_ 1 g, Tryptone 10 g, Beef Extract 3 g, agar 12 g. Before culturing on these plates, a 3-h pre-culture of *E. coli* B strain was centrifuged and re-suspended in 150 mM NaCl, 10 mM NaHCO_3_ or 10 mM KHCO_3_, respectively.

The dialysates of T4 phage were tested for plaque forming capability, assessed as described in detail in our previous publication [28].

## Abbreviations

AFM: atomic force microscopy
LV-FESEM: low-voltage field-emission scanning electron microscopy
DLS: dynamic light scattering
PDI: polydispersity index
LPS: lipopolysaccharides
GB: group behavior
QS: quorum sensing
